# Supplementary material for: Comparative Genomic Analysis of Three Pseudomonas Species Isolated from the Eastern Oyster (Crassostrea virginica) Tissues, Mantle Fluid, and the Overlying Estuarine Water Column
Source: Microorganisms. 2021 Feb 27;9(3):490. doi: 10.3390/microorganisms9030490 (PMC7996774; doi:10.3390/microorganisms9030490)
Supplement: Supplementary file 1 [file microorganisms-09-00490-s001.pdf]

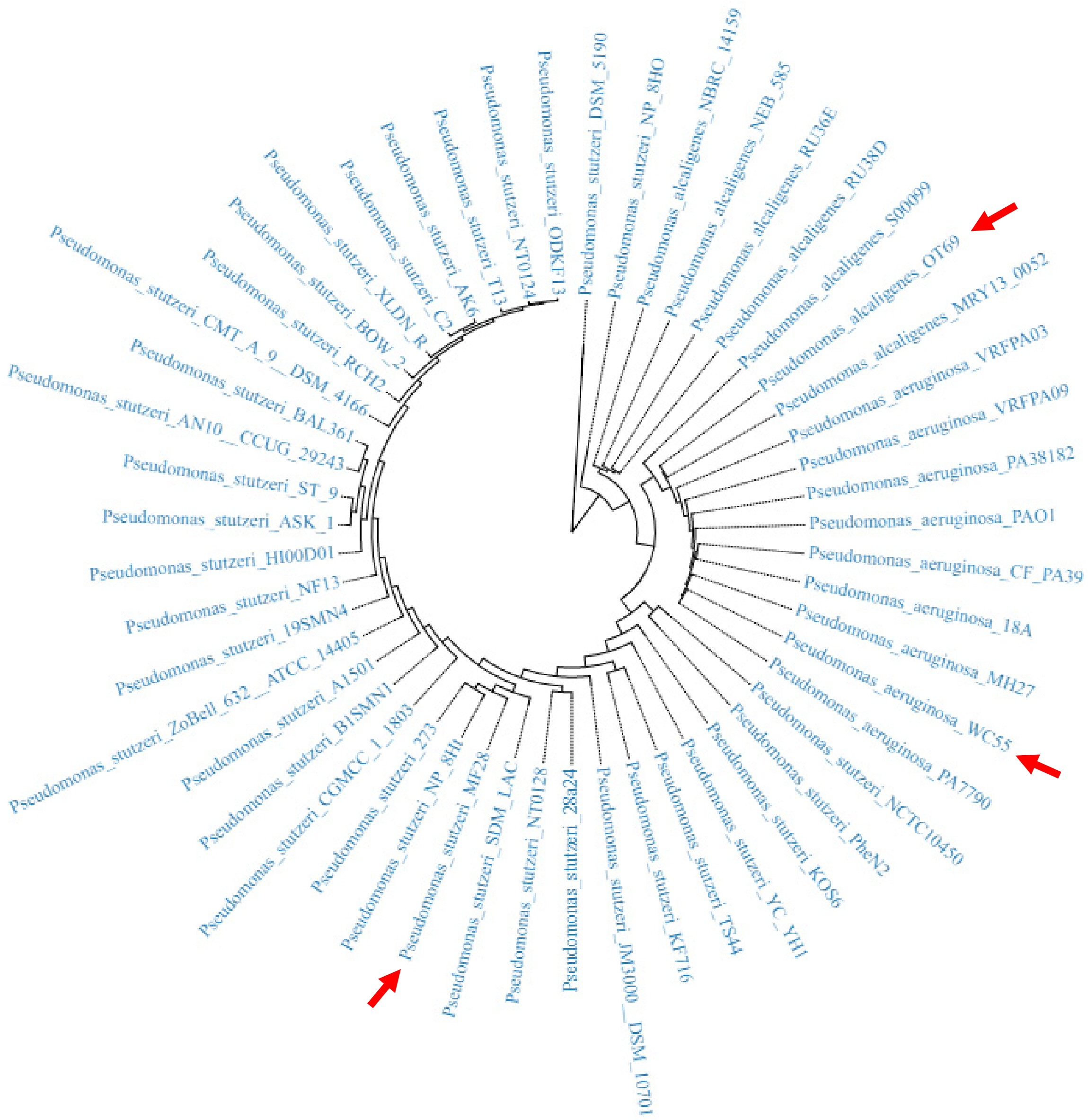

Fig. SI-1. Hierarchical clustering analysis of isolated *Pseudomonas* species. A total of 51 genomes were selected for this analysis which was run based on COG profiles using the img/er workflow.

A

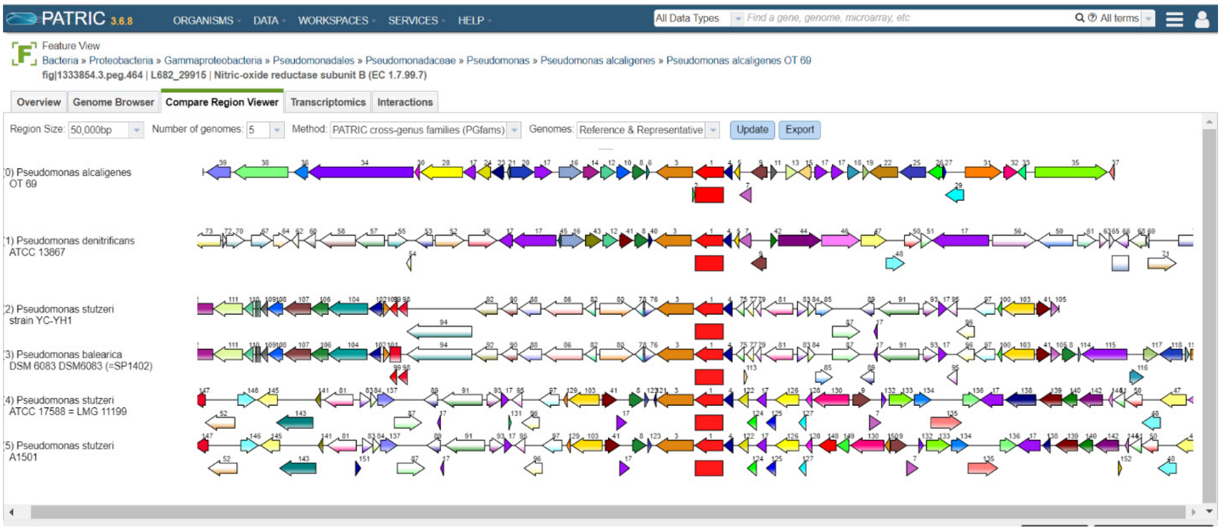
PATRIC 3.6.8
ORGANISMS
DATA
WORKSPACES
SERVICES
HELP
All Data Types
Find a gene, genome, microarray, etc
All terms
Feature View
Bacteria » Proteobacteria » Gammaproteobacteria » Pseudomonadales » Pseudomonadaceae » Pseudomonas » Pseudomonas alcaligenes » Pseudomonas alcaligenes OT 69
fig|1333854.3.peg.464 | L682_29915 | Nitric-oxide reductase subunit B (EC 1.7.99.7)
Overview
Genome Browser
Compare Region Viewer
Transcriptomics
Interactions
Region Size: 50,000bp
Number of genomes: 5
Method: PATRIC cross-genus families (PGfams)
Genomes: Reference & Representative
Update
Export
(0) Pseudomonas alcaligenes OT 69
(1) Pseudomonas denitrificans ATCC 13867
(2) Pseudomonas stutzeri strain YC-YH1
(3) Pseudomonas balearica DSM 6083 DSM6083 (=SP1402)
(4) Pseudomonas stutzeri ATCC 17588 = LMG 11199
(5) Pseudomonas stutzeri A1501

B

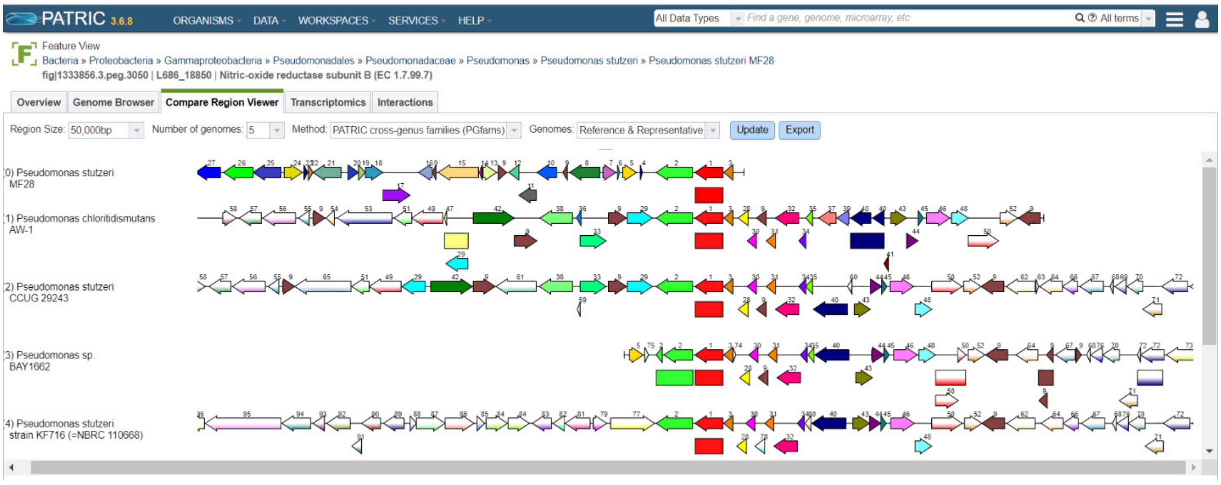
PATRIC 3.6.8
ORGANISMS
DATA
WORKSPACES
SERVICES
HELP
All Data Types
Find a gene, genome, microarray, etc
All terms
Feature View
Bacteria » Proteobacteria » Gammaproteobacteria » Pseudomonadales » Pseudomonadaceae » Pseudomonas » Pseudomonas stutzeri » Pseudomonas stutzeri MF28
fig|1333856.3.peg.3050 | L686_18850 | Nitric-oxide reductase subunit B (EC 1.7.99.7)
Overview
Genome Browser
Compare Region Viewer
Transcriptomics
Interactions
Region Size: 50,000bp
Number of genomes: 5
Method: PATRIC cross-genus families (PGfams)
Genomes: Reference & Representative
Update
Export
(0) Pseudomonas stutzeri MF28
(1) Pseudomonas chloritidismutans AW-1
(2) Pseudomonas stutzeri CCUG 29243
(3) Pseudomonas sp. BAY1662
(4) Pseudomonas stutzeri strain KF716 (=NBRC 110668)

C

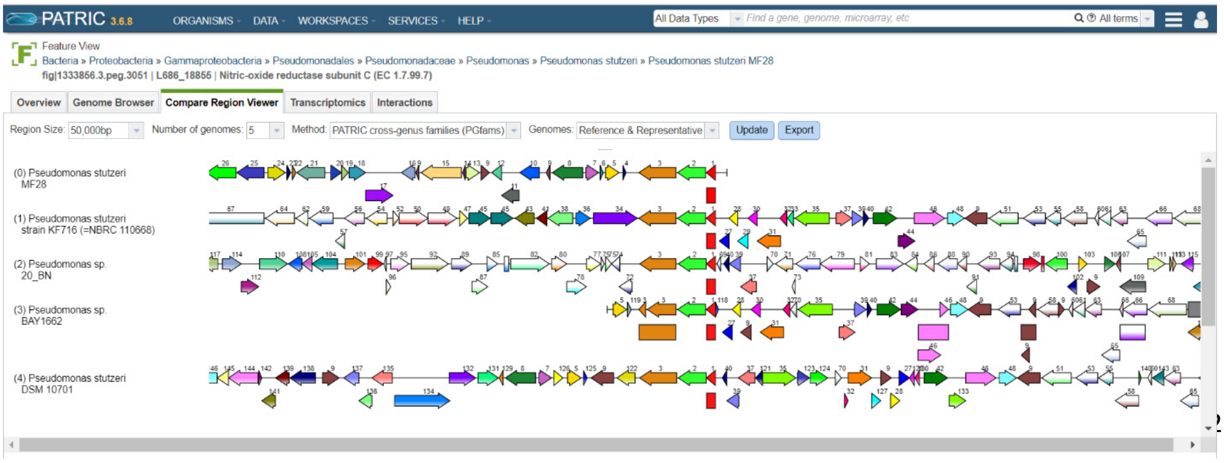
PATRIC 3.6.8
ORGANISMS
DATA
WORKSPACES
SERVICES
HELP
All Data Types
Find a gene, genome, microarray, etc
All terms
Feature View
Bacteria » Proteobacteria » Gammaproteobacteria » Pseudomonadales » Pseudomonadaceae » Pseudomonas » Pseudomonas stutzeri » Pseudomonas stutzeri MF28
fig|1333856.3.peg.3051 | L686_18855 | Nitric-oxide reductase subunit C (EC 1.7.99.7)
Overview
Genome Browser
Compare Region Viewer
Transcriptomics
Interactions
Region Size: 50,000bp
Number of genomes: 5
Method: PATRIC cross-genus families (PGfams)
Genomes: Reference & Representative
Update
Export
(0) Pseudomonas stutzeri MF28
(1) Pseudomonas stutzeri strain KF716 (=NBRC 110668)
(2) Pseudomonas sp. 20_BN
(3) Pseudomonas sp. BAY1662
(4) Pseudomonas stutzeri DSM 10701

D

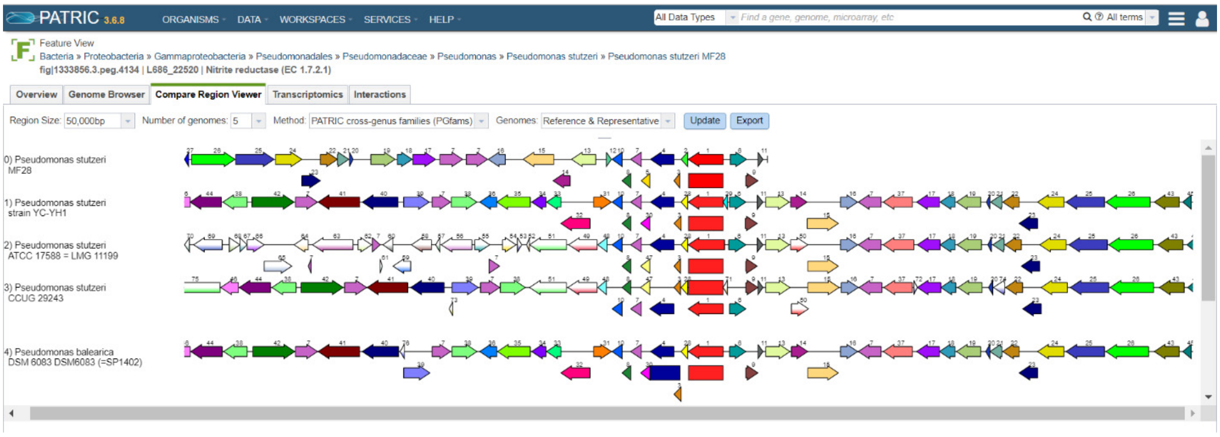
PATRIC 3.6.8
ORGANISMS DATA WORKSPACES SERVICES HELP
All Data Types
Find a gene, genome, microarray, etc
All terms
Feature View
Bacteria » Proteobacteria » Gammaproteobacteria » Pseudomonadales » Pseudomonadaceae » Pseudomonas » Pseudomonas stutzeri » Pseudomonas stutzeri MF28
fig|1333856.3.peg.4134 | L686_22520 | Nitrite reductase (EC 1.7.2.1)
Overview
Genome Browser
Compare Region Viewer
Transcriptomics
Interactions
Region Size: 50,000bp
Number of genomes: 5
Method: PATRIC cross-genus families (PGfams)
Genomes: Reference & Representative
Update
Export
0) Pseudomonas stutzeri MF28
1) Pseudomonas stutzeri strain YC-YH1
2) Pseudomonas stutzeri ATCC 17588 = LMG 11199
3) Pseudomonas stutzeri CCUG 29243
4) Pseudomonas balearica DSM 6083 DSM6083 (=SP1402)

E

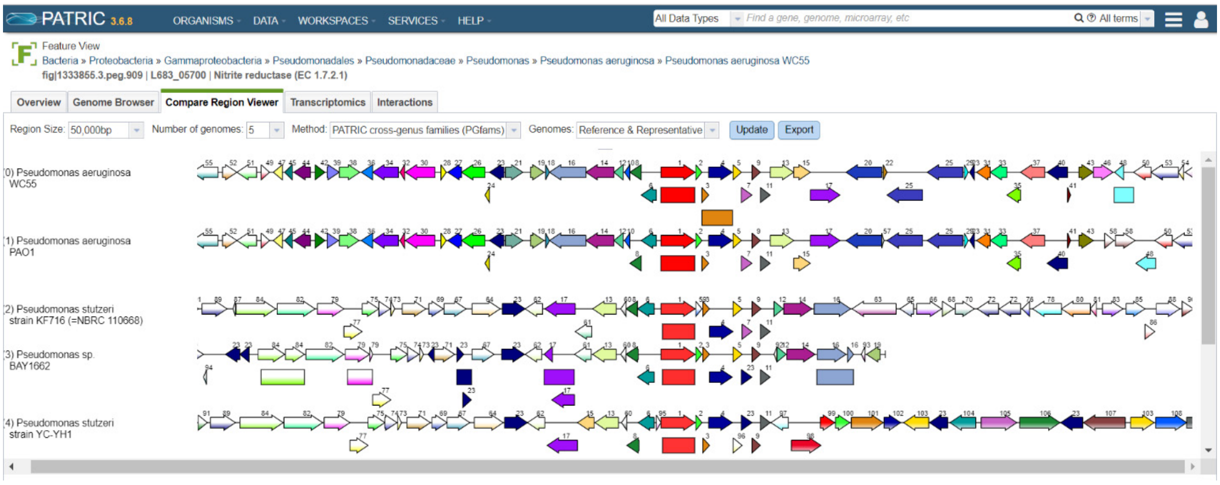
PATRIC 3.6.8
ORGANISMS DATA WORKSPACES SERVICES HELP
All Data Types
Find a gene, genome, microarray, etc
All terms
Feature View
Bacteria » Proteobacteria » Gammaproteobacteria » Pseudomonadales » Pseudomonadaceae » Pseudomonas » Pseudomonas aeruginosa » Pseudomonas aeruginosa WC55
fig|1333855.3.peg.909 | L683_05700 | Nitrite reductase (EC 1.7.2.1)
Overview
Genome Browser
Compare Region Viewer
Transcriptomics
Interactions
Region Size: 50,000bp
Number of genomes: 5
Method: PATRIC cross-genus families (PGfams)
Genomes: Reference & Representative
Update
Export
0) Pseudomonas aeruginosa WC55
1) Pseudomonas aeruginosa PAO1
2) Pseudomonas stutzeri strain KF716 (=NBRC 110668)
3) Pseudomonas sp. BAY1662
4) Pseudomonas stutzeri strain YC-YH1

F

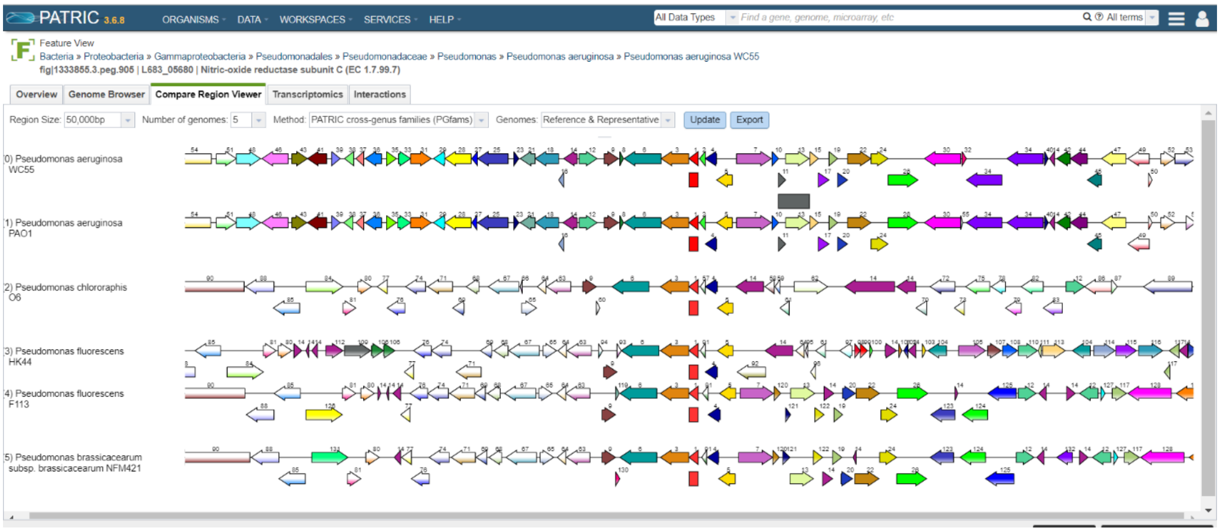
PATRIC 3.6.8
ORGANISMS DATA WORKSPACES SERVICES HELP
All Data Types
Find a gene, genome, microarray, etc
All terms
Feature View
Bacteria » Proteobacteria » Gammaproteobacteria » Pseudomonadales » Pseudomonadaceae » Pseudomonas » Pseudomonas aeruginosa » Pseudomonas aeruginosa WC55
fig|1333855.3.peg.905 | L683_05680 | Nitric-oxide reductase subunit C (EC 1.7.99.7)
Overview
Genome Browser
Compare Region Viewer
Transcriptomics
Interactions
Region Size: 50,000bp
Number of genomes: 5
Method: PATRIC cross-genus families (PGfams)
Genomes: Reference & Representative
Update
Export
0) Pseudomonas aeruginosa WC55
1) Pseudomonas aeruginosa PAO1
2) Pseudomonas chlororaphis O6
3) Pseudomonas fluorescens HK44
4) Pseudomonas fluorescens F113
5) Pseudomonas brassicacearum subsp. brassicacearum NFM421

G

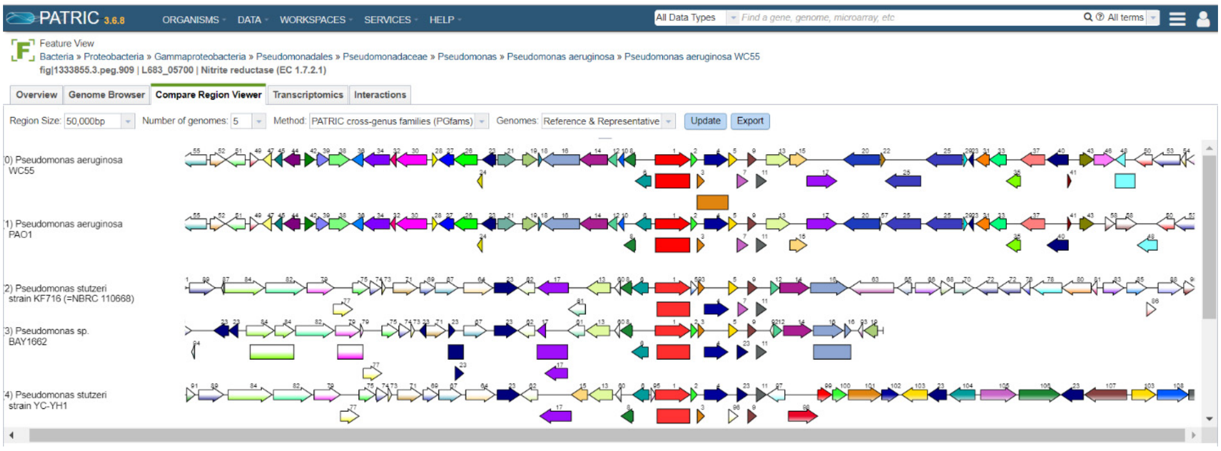

Fig. SI-2. Operonic structures of key denitrification genes identified in *Pseudomonas alcaligenes* OT69, *Pseudomonas stutzeri* MF28 and *Pseudomonas aeruginosa* WC55, relative to their closest relatives. Shown are nitric-oxide reductase subunit B in strain OT69 (2A); nitric-oxide reductase subunit B, subunit C and a nitrite reductase in strain MF28 (2B-D); strain WC55 also possessed these genes, which are the nitric-oxide reductase subunit B, subunit C and a nitrite reductase (2E-G), respectively.

A

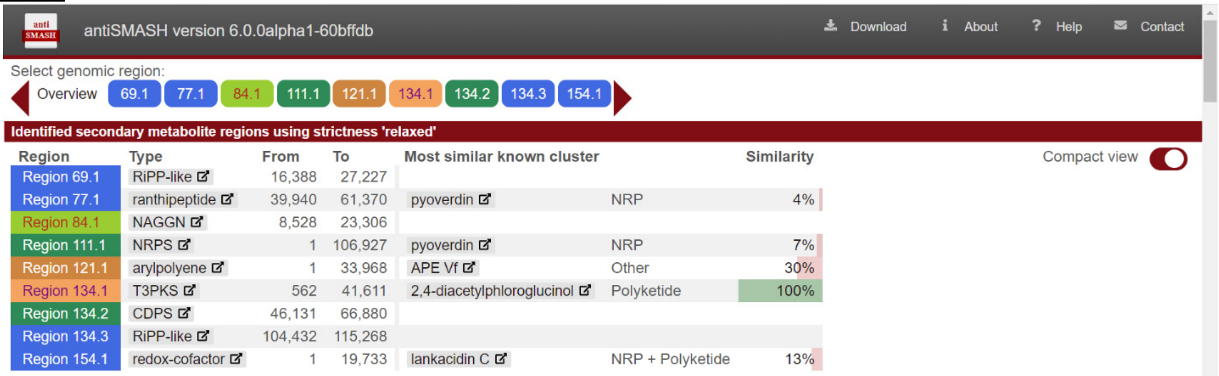

Region	Type	From	To	Most similar known cluster		Similarity
Region 69.1	RiPP-like	16,388	27,227			
Region 77.1	ranthipeptide	39,940	61,370	pyoverdin	NRP	4%
Region 84.1	NAGGN	8,528	23,306			
Region 111.1	NRPS	1	106,927	pyoverdin	NRP	7%
Region 121.1	arylpolyene	1	33,968	APE Vf	Other	30%
Region 134.1	T3PKS	562	41,611	2,4-diacetylphloroglucinol	Polyketide	100%
Region 134.2	CDPS	46,131	66,880			
Region 134.3	RiPP-like	104,432	115,268			
Region 154.1	redox-cofactor	1	19,733	lankacidin C	NRP + Polyketide	13%

B

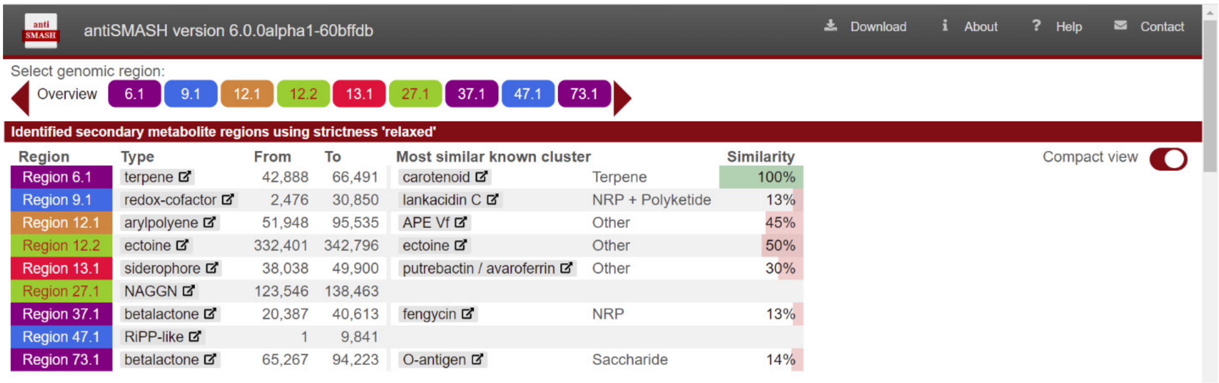

Region	Type	From	To	Most similar known cluster		Similarity
Region 6.1	terpene	42,888	66,491	carotenoid	Terpene	100%
Region 9.1	redox-cofactor	2,476	30,850	lankacidin C	NRP + Polyketide	13%
Region 12.1	arylpolyene	51,948	95,535	APE Vf	Other	45%
Region 12.2	ectoine	332,401	342,796	ectoine	Other	50%
Region 13.1	siderophore	38,038	49,900	putrebactin / avaroferrin	Other	30%
Region 27.1	NAGGN	123,546	138,463			
Region 37.1	betalactone	20,387	40,613	fengycin	NRP	13%
Region 47.1	RiPP-like	1	9,841			
Region 73.1	betalactone	65,267	94,223	O-antigen	Saccharide	14%

C

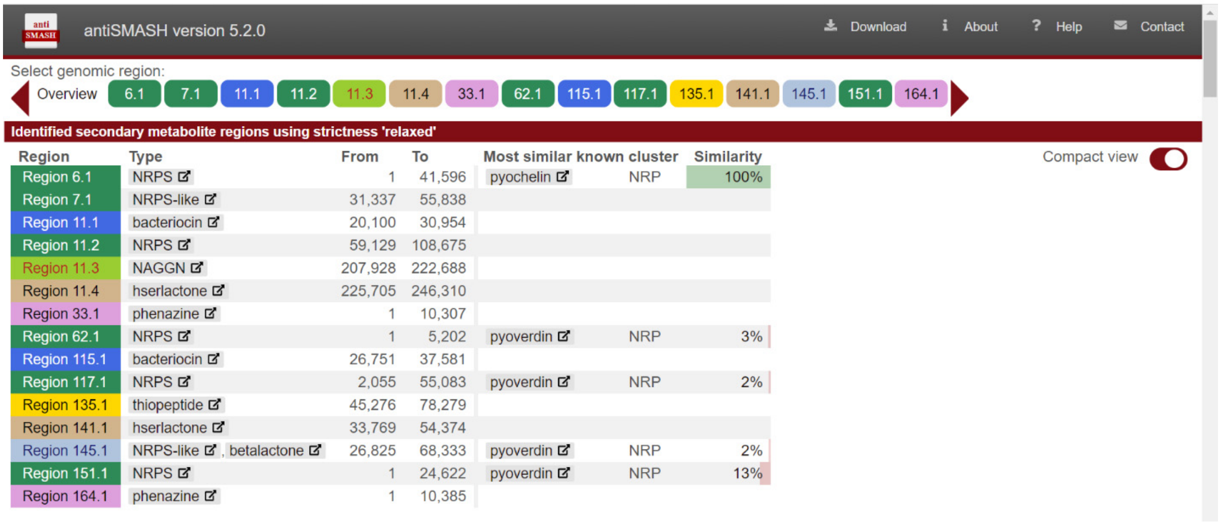

Region	Type	From	To	Most similar known cluster		Similarity
Region 6.1	NRPS	1	41,596	pyochelin	NRP	100%
Region 7.1	NRPS-like	31,337	55,838			
Region 11.1	bacteriocin	20,100	30,954			
Region 11.2	NRPS	59,129	108,675			
Region 11.3	NAGGN	207,928	222,688			
Region 11.4	hserlactone	225,705	246,310			
Region 33.1	phenazine	1	10,307			
Region 62.1	NRPS	1	5,202	pyoverdin	NRP	3%
Region 115.1	bacteriocin	26,751	37,581			
Region 117.1	NRPS	2,055	55,083	pyoverdin	NRP	2%
Region 135.1	thiopeptide	45,276	78,279			
Region 141.1	hserlactone	33,769	54,374			
Region 145.1	NRPS-like , betalactone	26,825	68,333	pyoverdin	NRP	2%
Region 151.1	NRPS	1	24,622	pyoverdin	NRP	13%
Region 164.1	phenazine	1	10,385			

Fig. SI-3. Biosynthetic gene cluster analysis performed on isolated *Pseudomonas* species. Shown are A, *Pseudomonas alcaligenes* OT69; B, *Pseudomonas stutzeri* MF28 and C, *Pseudomonas aeruginosa* WC55, respectively.

A

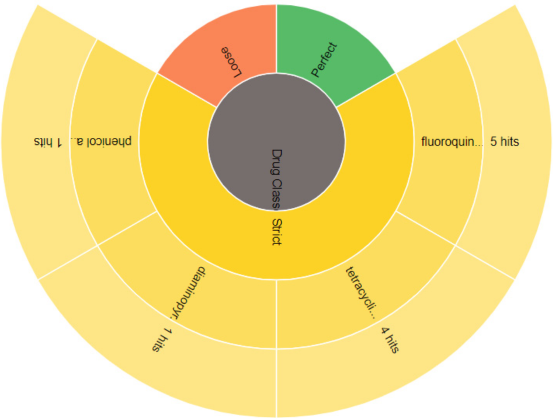
Drug Class
Strict
Loose
Perfect
fluoroquin... 5 hits
tetracycli... 4 hits
diaminopyr... 1 hits
phenicol a... 1 hits

B

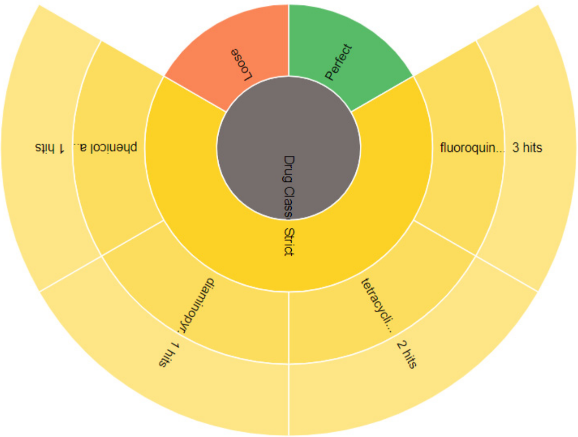
Drug Class
Strict
Loose
Perfect
fluoroquin... 3 hits
tetracycli... 2 hits
diaminopyr... 1 hits
phenicol a... 1 hits

C

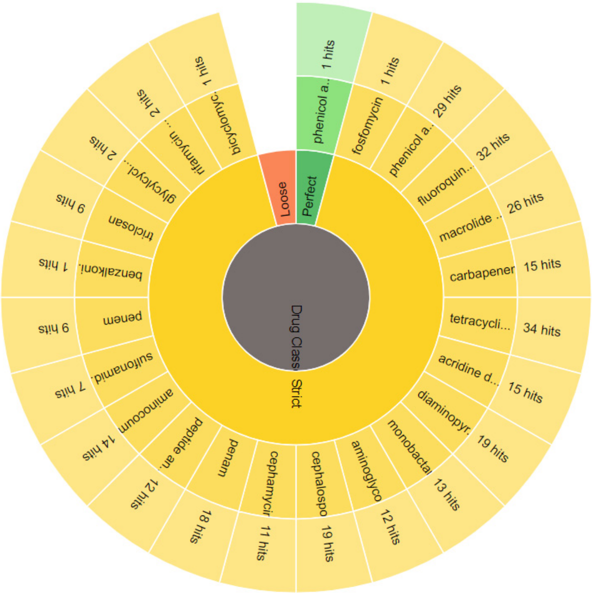
Drug Class
Strict
Loose
Perfect
phenicol a... 1 hits
fosfomycin 1 hits
phenicol a... 29 hits
fluoroquin... 32 hits
macrolide ... 26 hits
carbapener 15 hits
tetracycli... 34 hits
acridine d... 15 hits
diaminopyr... 19 hits
monobactal 13 hits
aminoglyco 12 hits
cephalospo 19 hits
cephamycir 11 hits
penam 18 hits
peptide an... 12 hits
aminocoum... 14 hits
sulfonamid... 7 hits
penem 9 hits
benzalkoni... 1 hits
triclosan 9 hits
bicyclomyc... 1 hits

Fig. SI-4. Evaluation of the resistome or the suite of antimicrobial resistance genes within the genomes of the three isolated pseudomonads using the CARD pipeline. Shown are A, *Pseudomonas alcaligenes* OT69; B, *Pseudomonas stutzeri* MF28 and C, *Pseudomonas aeruginosa* WC55, respectively.
